# Health Technology Assessment of Intensive Care Ventilators for Pediatric Patients

**DOI:** 10.3390/children8110986

**Published:** 2021-11-01

**Authors:** Martina Andellini, Francesco Faggiano, Sergio Giuseppe Picardo, Giuseppina Testa, Daniela Perrotta, Roberto Bianchi, Federico Nocchi, Carlo Capussotto, Elena Bassanelli, Pietro Derrico, Nicola Pirozzi, Leandro Pecchia, Matteo Ritrovato

**Affiliations:** 1 HTA Unit, Bambino Gesù Children’s Hospital, IRCCS, 00165 Rome, Italy; francesco.faggiano@opbg.net (F.F.); matteo.ritrovato@opbg.net (M.R.); 2 Department of Anesthesia and Critical Care, Bambino Gesù Children’s Hospital, IRCCS, 00165 Rome, Italy; sgiuseppe.picardo@opbg.net (S.G.P.); daniela.perrotta@opbg.net (D.P.); roberto.bianchi@opbg.net (R.B.); 3 Paediatric Cardiac Anesthesia and Intensive Care Unit, Bambino Gesù Children’s Hospital, IRCCS, 00165 Rome, Italy; giuseppina.testa@opbg.net; 4 Clinical Engineering Department, Bambino Gesù Children’s Hospital, IRCCS, 00165 Rome, Italy; federico.nocchi@opbg.net (F.N.); carlo.capussotto@opbg.net (C.C.); 5 HTA and Safety Research Unit, Scientific Directorate, Bambino Gesù Children’s Hospital, IRCCS, 00165 Rome, Italy; elena.bassanelli@opbg.net (E.B.); pietro.derrico@opbg.net (P.D.); 6 Paediatric Emergency Department, Bambino Gesù Children’s Hospital, IRCCS, 00165 Rome, Italy; nicola.pirozzi@opbg.net; 7 School of Engineering, University of Warwick, Coventry CV4 7AL, UK; L.Pecchia@warwick.ac.uk

**Keywords:** intensive care ventilator, intensive care unit, pediatric, HTA, hospital decision making, multi criteria decision analysis

## Abstract

This paper is aimed at addressing all the critical aspects linked to the implementation of intensive care ventilators in a pediatric setting, highlighting the most relevant technical features and describing the methodology to conduct health technology assessment (HTA) for supporting the decision-making process. Four ventilator models were included in the assessment process. A decision-making support tool (DoHTA method) was applied. Twenty-eight Key Performance Indicators (KPIs) were identified, defining the safety, clinical effectiveness, organizational, technical, and economic aspects. The Performance scores of each ventilator have been measured with respect to KPIs integrated with the total cost of ownership analysis, leading to a final rank of the four possible technological solutions. The final technologies’ performance scores reflected a deliver valued, contextualized, and shared outputs, detecting the most performant technological solution for the specific hospital context. HTA results had informed and supported the pediatric hospital decision-making process. This study, critically identifying the pros and cons of innovative features of ventilators and the evaluation criteria and aspects to be taken into account during HTA, can be considered as a valuable proof of evidence as well as a reliable and transferable method for conducting decision-making processes in a hospital context.

## 1. Introduction

Mechanical ventilation has been considerably progressing over the last 20 years, thanks to the development of more efficient and sophisticated technical features. It has gradually led to the development of different ventilation modes that had significantly improved the clinical effectiveness of ventilation techniques and patient–ventilator synchrony [1], leading in the meantime to reduction of time spent on ventilation, staff workload, and intensive care unit (ICU) costs [2,3]. Even if the variety of currently available ventilation modes and technical features are a great chance to solve respiratory failures, health professionals need to be aware about the technology and the risks that they could bring to the system [4].

For this reason, a comprehensive evaluation of the technical characteristics of ICU ventilators results is pivotal to understand the strengths and weaknesses of each ventilator, particularly because the ventilators’ efficacy provided by manufacturers may be significantly different from the clinical effectiveness in a real clinical setting.

The complexity of pediatric ventilation is firstly linked to the heterogeneity of pediatric population in terms of age and weight. Variable size of patients ranges from premature patients below 1 kg to adolescents weighing 70 kg. For this reason, the heterogeneity in patients’ age and size should be carefully considered during an evaluation process. Several challenges in the management of mechanically ventilated children have emerged over last years, especially in the optimization of patients-ventilator interaction to prevent ventilator-induced injuries and in the use of non-invasive ventilation (NIV) [5].

For instance, to prevent ventilator-induced injury, the innovative ventilator strategies refer to low tidal volume (pre-set range for both neo-natal and pediatric modes) and high level of positive end-expiratory pressure (PEEP) [6,7,8,9,10].

Moreover, to optimize and personalize pediatric ventilation, recent studies suggest monitoring the transpulmonary pressure and capnography [11,12,13,14].

Early identification of weaning readiness is another current challenge in the reduction of pediatric ventilator-induced complications. In fact, the development of the closed-loop system (CLS) optimizes ventilatory support according to patients’ specific needs [15].

Finally, during the last decades, the indication for NIV in pediatric patients has been significantly growing [16], especially for the spread of high-flow nasal cannula that has been widely adopted in pediatric clinical practice because of its clinical benefits in a pediatric population [5].

In addition to the technological complexity and the heterogeneity of patients, the heterogeneity of ventilator machines is another aspect to be carefully analyzed. Moreover, the presence of several ICUs in the same hospital, with high care complexity and with different medical specialties, makes the theme of technologies’ heterogeneity crucial. Major issues are associated with the learning curve of healthcare professionals in transferring the theory into clinical practice. The learning curve theory is usually based on homogeneous products. The higher the heterogeneity of intensive care ventilator models within the same clinical setting is, the higher the difficulties in covering the clinical needs.

Another critical aspect associated with intensive care ventilation are costs. Recent years have been characterized by a significant increase of healthcare costs [17,18]. Expenses of the Intensive Care unit (ICU) usually cover up to 20% of the overall hospital costs [19] especially when considering those patients who require prolonged mechanical ventilation [20]. All the critical aspects mentioned above make the selection of the intensive care ventilator model that best fits with the specific healthcare setting needs difficult.

The introduction of a health technology in a hospital context is aimed at contextualizing both evidence and decisions. The boundary conditions of the specific healthcare setting will affect the decision-making process and the organizational aspects, for instance, will play a crucial role in the final decision. Considering the high complexity of the decision-making process for ICU ventilators selection, and taking advantage of the renewal plan for the intensive care ventilators, the hospital management of Bambino Gesù Children’s Hospital requested a detailed Health Technology Assessment (HTA) study aiming at the replacement of the intensive care ventilators in three hospital’s departments.

The aim of this study, indeed, was to provide an exhaustive overview of the assessment criteria that should be considered when comparing the performances of different ventilators models and to show an effective and reliable evaluation method.

The paper describes in detail the evaluation process that has been conducted within our hospital context offering a clear examination of the ventilators’ impact on the possible risk and clinical benefits for patients, the entire hospital organization, and the hospital budget. Thanks to the HTA process, which represents a multidisciplinary process and a method of evidence synthesis, it was possible to determine the ICU ventilators’ main technical characteristics, to measure their performances, to analyze the pros and the cons of different ventilators’ models, and to sum up all the information gathered and elaborated in an evidence-based final recommendation, actively supporting the complex decision-making process.

Different aspects such as clinical effectiveness, safety, costs, and technical and organizational aspects of the ventilators were analyzed. More specifically, the clinical benefits strictly related to the technological advances in mechanical ventilation have been thoroughly addressed and assessed during the analysis of clinical effectiveness and safety aspects, by means also of critical review of existing scientific literature. According to the literature, complex decision-making processes have been usually carried out through Multi-Criteria Decision Analysis (MCDA) method in several fields, even though very few applications to guide resource allocation decisions in health care have been recorded, so far. As MCDA results in an effective and reliable tool able to identify the criteria and their weights for priority setting, resulting in a rank ordering of interventions, we decided to adopt MCDA as an integral part of the HTA evaluation [21], as an analytical quantitative instrument focused on supporting the decision-making process between alternative products.

## 2. Materials and Methods

As the HTA is a multidisciplinary and multidimensionality process, a working group, composed by professionals with different professional skills, was established to identify all the pertinent aspects to be analyzed in the assessment of different ventilator systems. The working group involved eighteen professionals: twelve medical doctors; five Biomedical Engineers; one Health Economist. Table 1 illustrates the different responders’ professional profile and hospital department they are addressed to.

**Figure 1:**
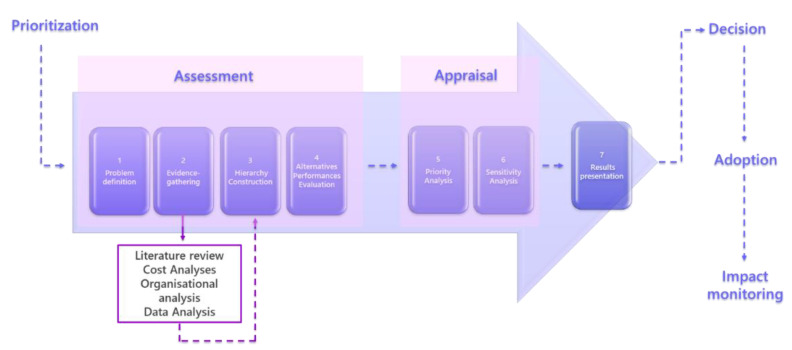
DoHTA method [21].

The HTA process was carried out integrating the evaluation conducted following the EUnetHTA CoreModel© (http://www.htacoremodel.info/BrowseModel.aspx, accessed on 10 September 2021), as a guideline for HTA processes [21,22,23], and MCDA by using the Analytic Hierarchy Process (AHP) [24,25,26,27], as outlined in Decision-oriented Health Technology Assessment (Do-HTA) method [21] and graphically schematized in Figure 1.

This method proposes to organize the collected evidence of the selected technology and the consequences of its use in different Key Performance Indicators (KPIs), which are arranged in a hierarchical decision structure (as required for AHP method). The integration of the analytical approach (AHP) within the qualitative assessment tool (EunetHTA), allows the quantifying of opinions and transforming them into a coherent decision model, assessing all factors and their interactions in a decision domain, leading one to define a final rank of several health interventions. The idea is that a decision problem can be decomposed into a hierarchy of more sub-problems each of which can be measured and analyzed independently. AHP mathematical process using pairwise comparisons, eigenvector method for deriving weights, and a method to verify the “consistency” of judgments, is able to integrate all KPIs evaluation in a final numerical result, which represents the alternatives’ relative ability to achieve the decision goal [24,25]. The whole process and the computational steps are described in details in a previous publication and it is under the name of DoHTA [21]. This method is characterized by seven steps as graphically represented in Figure 1.

The first phase of the HTA process is aimed at defining and detailing the decision problem. This project was commissioned by the hospital management for the renewal of the ventilators of three hospital’s departments. The decision problem, indeed, was to select the ventilator model that best fits with the hospital needs. Within the variety of models and manufactures currently available on the market, the working group drew up the minimum requirements that the ventilators should have to be considered eligible for the assessment process. More specifically, all the ventilators’ models must meet the essential specifications for Pediatric and Neonatal Intensive Care Ventilators recommended by ECRI (https://www.ecri.org/, accessed on 10 September 2021). More specifically, each model must be approved for neonatal and pediatric patients, must have a tidal volume (the volume of gas inhaled and exhaled during one respiratory cycle; models should be capable of providing volume ventilation) range of at least between 1 mL and 1000 mL, must have leak compensation function (a delivered flow to compensate for variable leaks) to maintain the synchrony between the patient and the ventilator, because most infants have leaks due to uncuffed airways, and it must have a non-invasive ventilation mode. Moreover, physicians required that the models should have a touch screen display with a size less than 21 because of the specific environment needs. Finally, four ventilators models that meet the inclusion criteria were selected as alternatives for the assessment process. The HTA process was carried out involving the three hospital departments that took part in the renewal plan: the Intensive Care Unit (ICU), the Emergency Department, and the Cardiac ICU. Each ventilator model was tested in each department by the medical doctors involved in the study for a period of up to three months. After the free trial period, health professionals were asked to provide a score related to the performances of each ventilator model that the professionals observed and measured during the trial period.

The entire HTA process will be shown as a case study calling the different ventilators’ models Model 1, Model 2, Model 3, and Model 4.

After defining the decision problem, a preliminary study was conducted to acquire a general overview of the subject and to define the main criteria representing discriminant assessment factors between the technologies under evaluation and the state of the art.

The main evaluation criteria (Domains) were identified, by the working group, among those singled out by the EUnetHTA Core Model© [22,23] that represents the European guideline for each HTA process. Following the EUnetHTA Core Model, each technological innovation could be analyzed according to nine domains: the current use of the technology, safety, clinical effectiveness, costs, social, ethical, legal, technical, and organizational aspects.

Each domain of the EunetHTA Core Model is composed of different topics that represent more specific aspects within the domain and can, in turn, be described by one or more issues. An issue underlies a specific factor within a topic. The combination of a domain, topic, and issue defines one assessment element, which defines a piece of information that describes the technology or the consequences or implications of its use or any other aspect relevant for the assessment.

Each assessment element defines a question whose answer may bring a piece of knowledge that can potentially be translated into an indicator of the decisional structure. To integrate the AHP method, the assessment elements are translated in the form of criteria or sub-criteria, which we called KPIs. This means that selected topics and issues are finally translated into a list of specific and narrow elements.

In this way, each evaluation domain was defined throughout several KPIs, in order to detail the decision problem and describe which aspect of each evaluation area the working group intends to take into account.

To outline a clear, detailed, and proper definition of the indicators, answering the questions of the selected assessment elements, a general literature search was carried out aimed at identifying the detailed indicators through which the intensive care ventilator models could be evaluated for each domain (details are presented in Appendix B).

According to AHP, the KPIs identified were arranged in a hierarchy decision tree. More specifically, the “goal of the decision” was split into the main evaluation criteria (i.e., the domains), each one being divided in turn into KPIs, covering all the pertinent aspects to be analyzed in the assessment of the different ventilators’ systems. All indicators and domains were judged in terms of performances’ values (Assessment phase) and relative weights (Appraisal phase) through the AHP method (Figure 1). After the ventilators’ free trial period, each member of the working group provided a score related to the ventilators’ performances basing on the scientific evidence that emerged during the trial period and the technical documentation provided by each manufacturer. More specifically, the absolute performances scores of each ventilator model were attributed looking at the best performant ventilator with respect to the specific KPI [24,26,28].

Moreover, members of the working group assigned to each element of the decision tree a relative weight basing on their experience, on the scientific evidence that emerged during the trial period and on the boundary conditions of the specific clinical setting in which the ventilators will be implemented. More specifically, following the AHP method, through pairwise comparison, for each pair of elements of the decision tree, each professional attributed a relative rating answering to the questions about the elements’ relative importance. AHP method allows one to convert the qualitative judgements from pairwise comparisons to consistent numerical values [24,25,28].

**Figure A1:**
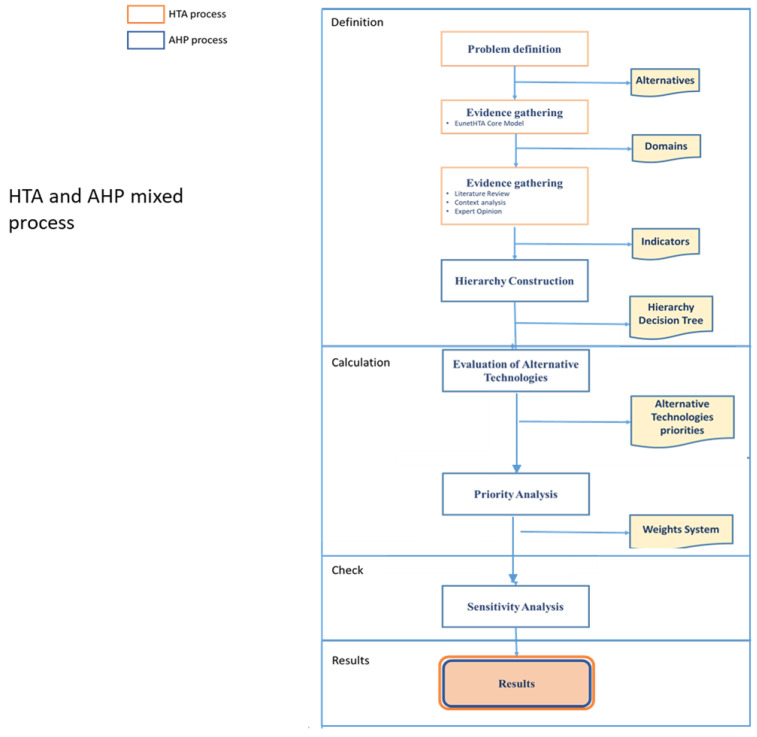
The integration of the analytical approach (AHP) within the qualitative assessment tool (EunetHTA) allows the quantifying opinions and transforming them into a coherent decision model, assessing all factors and their interactions in a decision domain, leading one to define a final rank of several health interventions. The idea is that a decision problem can be decomposed into a hierarchy of more sub-problems, each of which can be measured and analyzed independently. The AHP mathematical process using pairwise comparisons, eigenvector method for deriving weights, and a method to verify the “consistency” of judgments, is able to integrate all KPIs evaluation in a final numerical result, which represents the alternatives’ relative ability to achieve the decision goal.

Results of the AHP method reveal the global weight of each KPI, calculated aggregating the derived weights’ system from each professional interviewed. This indicates the relevance of each assessment element within the overall evaluation. Moreover, the absolute performance values of each KPI were then weighted by the relative domains weight, in order to obtain the alternative technologies’ scores and the final global rank of the four ventilators models. The whole process, the computational steps, and the aggregation method are graphically shown in Appendix A (Figure A1) and are described in detail in a previous publication, under the name DoHTA [21], and in AHP related literature [24,25,26,27,28].

### 2.1. Economic Evaluation

The economic evaluation was carried out considering all business proposals and comparing all purchase costs. The analysis of Total Cost of Ownership (TCO) [29] has been carried out to understand the relevant cost of buying a particular good from a particular supplier. It is a financial estimation focused on helping managers to determine direct and indirect costs of a product or system. This analysis informed the cost and economic evaluation domain results.

### 2.2. Sensitivity Analysis

The AHP weighting process as per the definition of the system or prioritization is intrinsically subjective. However, the judgements of experts of the working group are based on scientific evidence, measurements (where possible), and on the boundary conditions of the clinical setting in which the technology is going to be introduced. Moreover, to test the stability and the robustness of the alternatives’ ranking, a sensitivity analysis was performed.

The analysis was conducted comparing the models with the highest performance value, to identify the elements representing the source of uncertainty and to determine the impact of this variability on the stability of the assessment results. It was applied to both weights and performance system. Sensitivity analysis was carried out by calculating the minimum changes on values (changes on the experts’ judgements) of each criterion needed to reverse the final ranking of alternative technologies [30].

## 3. Results

### 3.1. Evidence Gathering

Through the information gathered from the general literature search, integrated with the information from manufactures, the working group selected 28 KPIs, which represent the main factors for discriminating the technologies under evaluation.

### 3.2. Hierarchy Construction

The working group decided to include in the analysis the domains that are able to highlight the differences and that represent discriminant factors among the technologies under evaluation. Domains for which the four ventilators’ models perform equally were not considered for the analysis.

More specifically, the working group decided to include five domains in the analysis: safety, clinical aspects, costs and economic evaluation, technical characteristics, organizational aspects, whereas legal, social, and ethical domains were not considered, as often happens in Hospital-Based HTA processes (http://www.adhophta.eu/sites/files/adhophta/media/adhophta_handbook_website.pdf, accessed on 10 September 2021), because the level, the type of analysis, and the consequent decisions do not imply any substantial impact on these domains.

Each domain was then described by a subgroup of the 28 KPIs previously identified.

**Figure 2:**
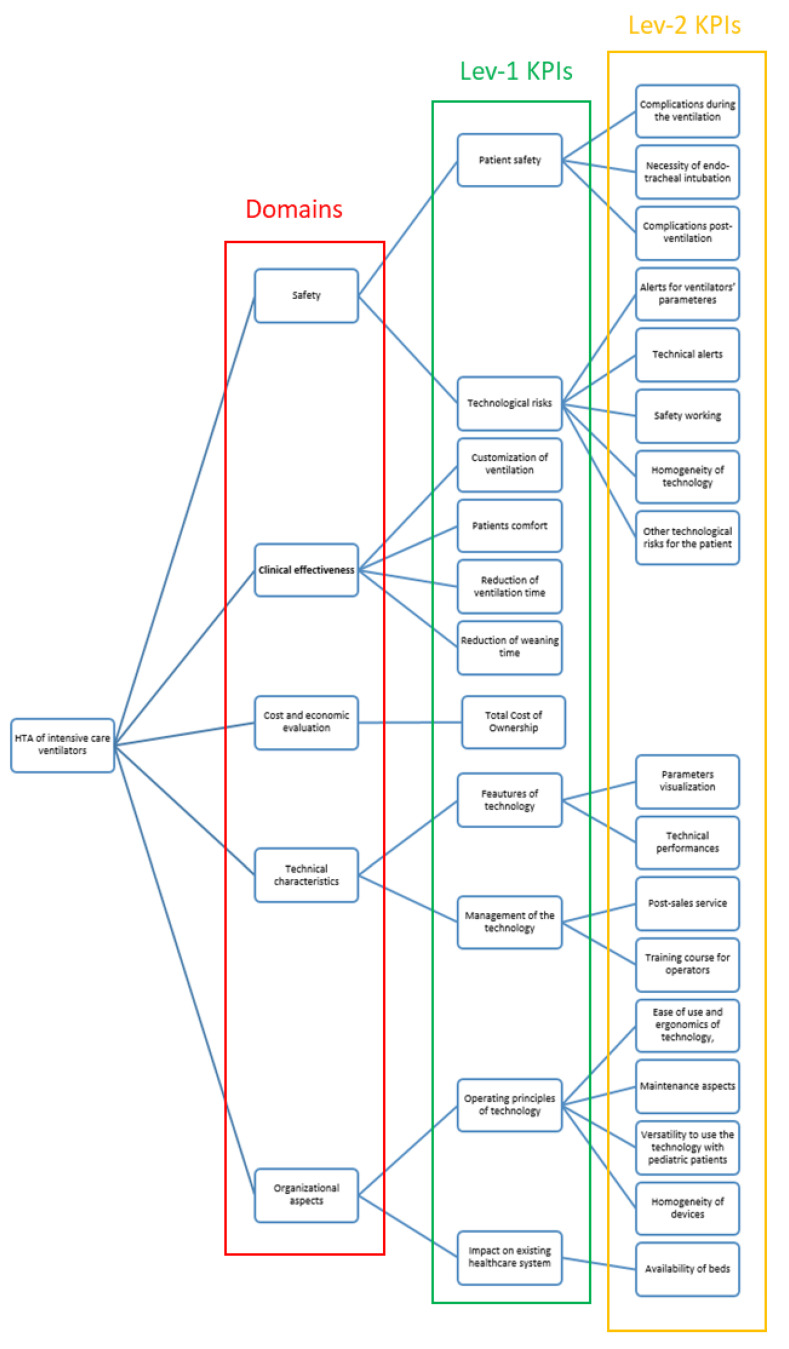
Hierarchical decision tree (Hierarchy construction): domains, Level 1, and Level 2 key performance indicators.

The 28 KPIs were arranged in a hierarchical decision tree (Figure 2) as Lev-1 and Lev-2 KPIs.

Moreover, as the aim of this HTA process was to compare the performances of four ventilators models, detailed technical specifications should be defined to discriminate the best performer. For this reason, each Lev-2 KPI was then described by several technical specifications (Lev-3 KPIs), each of which is measurable and intrinsically provides an effect within the related domain. Lev-3 KPIs, defined by the working group, is a list of technical specifications (gathered from those available on the market) that represents the “material” way a ventilator may fulfil the objectives listed in the Lev-2 KPIs.

The final list of the Lev-3 KPIs and their topological arrangement within the decision tree will inform the request for proposal to the four manufacturers. To give an example, it is believed that better complications rate (patient safety) might be reached if the ventilator’s model guarantees the minute-volume ventilation mode or if it provides the compliance and resistance monitoring system. Detailed information and description of Lev-1, -2, and -3 KPIs are shown in Table 2.

### 3.3. AHP Indicators’ Weights and Priorities

Ventilators models’ performances were compared among each other using as reference the Level-3 KPIs. Professionals, after testing the four ventilators’ models and assessing the scientific evidence that emerged during the trial period and the technical documentation provided by the manufacturers, for each Lev-3 KPI, for each ventilator model, assigned a score from 1 to 5. Through the AHP method [24,25], scores were than translated into percentage values.

Furthermore, for each element of the decision tree, a relative weight was assigned according to the AHP method [24,25].

**Figure 3:**
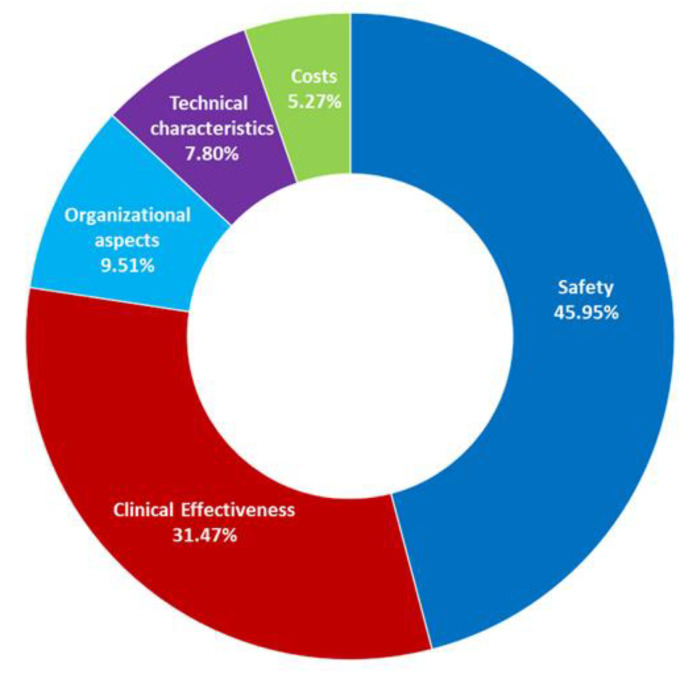
Ring plot illustrating the unified weights’ system (which represents the percentage level of importance of the various domains with respect to the overall evaluation) pertaining to the “domains” layer of the decision tree, as gathered from pairwise comparisons and mathematical calculations in accordance with the AHP method).

**Figure 4:**
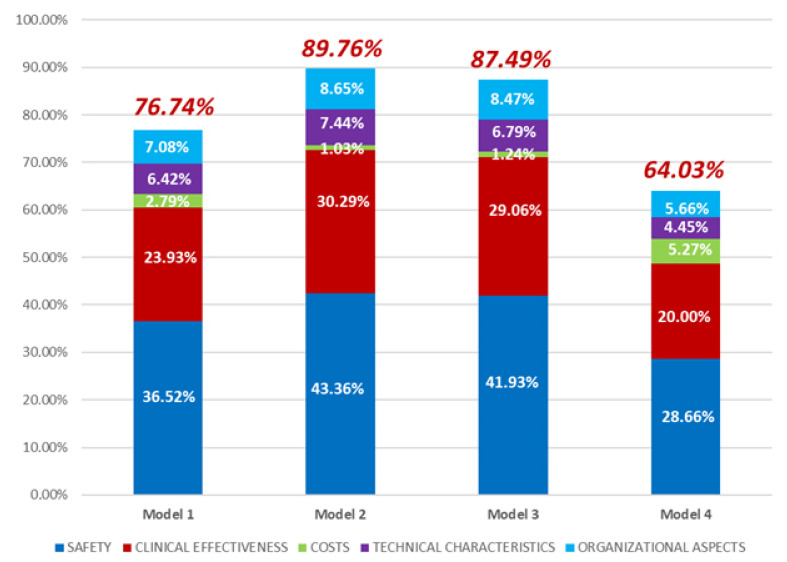
Histogram chart specifying the computed performance (global and per domain) of intensive care ventilators. The ventilators were compared with their alternatives with respect to every lowest indicator according with the AHP method.

The absolute performance values of each Level-3 KPI was then weighted by the relative domains weight calculated in the previous step, in order to obtain the alternative technologies’ scores and the final global rank of the four ventilators models. The whole process, the computational steps, and the aggregation method are graphically shown in Appendix A (Figure A1) and are described in detail in a previous publication, under the name DoHTA [21], and in AHP related literature [24,25,26,27,28]. AHP results are shown in Table 3 and graphically represented in Figure 3 and Figure 4. More specifically, the ring plot (Figure 3) represents the unified weights’ system considering the results of all the involved professionals’ judgements, pertaining to the “domains” layer of the decision tree, according to the mathematical calculations of the AHP method. More specifically, the ring plot represents the aggregation of the weights of KPIs assigned by each professional interviewed.

Results showed that the most important aspects to be considered when comparing different ventilators’ models are the safety and the clinical effectiveness, reaching, respectively, 45.95% and 31.47% of the total weight, followed by the organizational aspects (9.51%), technical characteristics (7.80%), and costs and economic evaluation (5.27%). Detailed information about each indicator weight is listed in the Table 3 (6th column). Detailed information about the calculation of these percentage values is shown in the AHP related literature [24,25].

More specifically, within the safety domain, the “patients’ safety” weights more than twice the “technological risks”. The “patient safety” KPI, indeed, covers 70% of the weight of the domain. It is most represented by the “complication during ventilation” which doubled the weight of “complication post ventilation”.

The “alert for ventilators’ parameters” represents the most critical KPI regarding the “technological risks”. The others KPIs (safety mechanisms, risk related to the heterogeneity of ventilators models, and the adverse events/recalls) assume more or less the same relevance within the evaluation.

The clinical effectiveness domain, which represents the second domain in order of importance, is described by a number of technical specifications which more or less equally affect the clinical outcomes such as the possibility to customize the ventilation, patients comfort related to the availability of non-invasive ventilation mode, the reduction of ventilation time and the weaning time.

The technical characteristics domain was mainly described by different aspects of the technology management and by a number of specifications emerged from technical documentation of different devices’ producers.

Regarding the organizational aspects, the impact that different operating principles of technologies have on the organization doubled the “impact with the existing healthcare systems”. More specifically, the former includes the analysis of the “ease of use and ergonomics of technology”, the “maintenance”, the “versatility to use the technology with pediatric patients”, and the availability of homogeneous technologies, compared to the latter which has been measured in terms of availability of beds where the technologies could be installed.

Finally, results on economic evaluation aimed to compare all business proposals considering all purchase costs and hypothesizing 10-year life-cycle cost estimate for each ventilator. Table 4 showed the comparison within the four models.

The histogram chart (Figure 4), instead, specifies the computed performance (global and per domain) of the different ventilator models (Models 1, 2, 3, and 4). According to AHP [24,25], performances were expressed as percentages and derived from the elaboration of the pairwise comparisons made by all professionals involved.

As shown in Figure 4, the Model 2 seems to be the best option comparing with the other Models. The final performance value of Model 2 has led 2.3% over Model 3 (89.76% vs. 87.49%) and 13% over Model 1 (89.76% vs. 76.74%). Table 3 (2nd, 3rd, 4th, 5th columns) gives detailed information about each indicator performance value for the four Models presented and about the relative weight of each element with respect of the overall evaluation (6th column).

Regarding the safety aspects, nevertheless, Model 2 seems to be safer than the others, reaching 42.36% performances score; it does not significantly differ from the Model 3 overall safety performances score (41.93%), whereas Model 1 and 4 performed worst.

It resulted that Model 2 provides several technical functions that, limiting the complication during ventilation, improve patient safety, including trigger mechanisms, apnea alarm, resistance and static dynamic compliance monitoring, lung recruitment tools (PV loops), higher number waveforms simultaneously displayed, and adjustable screen for ergonomics. It emerged that the necessity for endotracheal intubation is reduced if the ventilator model provides compensation for large leaks during NIV, trigger flow mechanism and tube compensations. All these aspects are better represented by Model 2, which is able to provide the best performances with respect of these technical characteristics. Model 1 provides control of optimal cuff pressure during the entire ventilation period, helping to prevent the complications post-ventilation such as ventilator associated pneumonia (VAP) and tracheal injuries.

To reduce the technological risks, Model 2 provides more programmable alarms than the other models as well as a system control for potentially dangerous involuntary changes to ventilation settings.

In relation to the clinical effectiveness, Figure 4 shows that Model 2 outperforms the other Models even though the performances score is quite like that reached by Model 3. Model 2 offers a better customization of ventilation providing a number of advanced ventilation modes and innovative technical characteristics aimed at reducing asynchronies, which are a frequent issue in ventilated patients. As the better performer, regarding the customization of the ventilation, Model 2 provides continuous and reliable measurement of static compliance of the lung and thorax, automatic trigger mechanism, lung recruitment tools to easily and safely perform lung recruitment maneuvers, the possibility to program frequency and amplitude of sigh breath function, etc. Model 2, throughout advances in variable Pressure support ventilation mode (PSV), improves synchrony and reduces the ventilation time. Model 3, instead, with automated weaning modes, provides the possibility to reduce weaning time.

The technical characteristics domain was described by a number of specifications that emerged from technical documentation of different devices’ manufacturers and by different aspects of the technology management. Even regarding this domain, Model 2 resulted in the best performer (Figure 4). In detail, as mentioned before, Model 2 provides more advanced technology features to make easier and more intuitive the visualization of information during ventilation, such as an adjustable screen and the possibility to simultaneously visualize more than one curve or loop. Model 2 also ensures high level accuracy and the availability of a wide measure range of monitored parameters (e.g., tidal volume, respiratory rate fraction of inspired oxygen, etc.). Moreover, regarding the post-sales service, while the manufacturer of Model 2 offers higher preventive maintenance frequency and continuous clinical and technical assistance than the others, the manufacturers of all four ventilator models offer the same conditions in terms of support services and training for biomedical technicians.

Organizational aspects analyze different aspects of the management of the technology and the impact of its introduction on the existing workflows.

Model 2 also performs better considering the ease of use and the ergonomics of the technology, providing some technical characteristics (i.e., adjustable screen, the measurement of percentage levels of oxygen, the calibration of flow and oxygen sensors, etc.) that make the ventilator more usable and enhance the human–machine interface aspects. Moreover, Model 2 is also the more versatile model for pediatric use, because of the wider range of tidal volume.

Finally, results on economic evaluation, as showed in Table 4, aimed to compare all business proposals considering all purchase costs and hypothesizing 10-year life-cycle cost estimate for each ventilator. From this analysis, it resulted that Model 4 was the best option with a total cost of ownership of about €30,742.66. The highest performance score, indeed, is attributed to Model 4 (the cheapest) with 5.27% in comparison to Model 1, 3 and 2 (2.79%, 1.24%, 1.03%, respectively) (Figure 4).

### 3.4. Sensitivity Analysis

Sensitivity analysis was carried out comparing the models with the highest final performances value to test the stability and reliability of the solution. The Model 2 seems to be the best option, even if its total performances score is very close to that of Model 3 (89.76% vs. 87.49%, respectively). The more similar the different models’ final scores are, the higher the probability to reverse the final solution. As the final performances value of Model 2 has led only 2.3% over Model 3, the sensitivity analysis was carried out between these two models.

Sensitivity analysis results on performances showed that to reverse the current ranking of the two different models, at least one of the following conditions has to be verified:

- A 3% reduction of Model 2 safety performance and a simultaneous 3% increase of Model 3 safety performance.
- A 5% reduction of Model 2 clinical effectiveness performance and a simultaneous 5% increase of Model 3 clinical effectiveness performance.

Results showed the instability of this solution. A minimum variation in the evaluation parameters’ final scores (3% in safety and 5% in clinical effectiveness) might reverse the final decision. It can result in a substantial equivalence between Model 2 and Model 3.

Results on weights, instead, showed that to reverse the current ranking of different models, a 3.5-fold increase of cost and economic evaluation weight and a simultaneous 14% reduction of the other dimensions’ weight should occur. It revealed, instead, a greater stability of the solution from the weight system point of view because such a level of importance to costs, instead of organizational or clinical effectiveness aspects, has been considered inappropriate by those responsible for the decision making process in our hospital.

## 4. Discussion

This project was commissioned by the hospital management for the renewal of all the hospital’s ventilators.

For these reasons, an HTA process was carried out in order to identify the best suitable model of critical care ventilator available on the market by comparing four different models.

The Do-HTA method [21] was used to yield a structured outcome able to support the decision-making process concerning the final decision on choosing the best technology to be adopted. Therefore, evidence gathered from the general literature search, from professionals’ expertise, and those collected during the trial period were integrated as results of the Do-HTA method.

An important point of this study was the identification of KPIs useful to compare the ventilator models and the construction of the decisional hierarchy tree. It provides and shares a general overview of the most relevant features to be considered in the assessment of intensive care ventilators for pediatric patients and relevant information about the detailed technical characteristics (Lev-3 KPIs) that provide effects within the related domain.

The main informative indicators we found were about safety, clinical effectiveness, technical characteristics, cost and economic evaluation, and organizational aspects. The safety and the clinical effectiveness have been defined as the most important domains within the assessment. They covered more than 75% weight of the total evaluation.

As regards the safety domain, the most important indicator into patient safety (Lev-1 KPI) is the “complication during the ventilation”, followed by the “complication post-ventilation”, and “necessity of endo-tracheal intubation”.

A recent study showed that an inappropriate use of ventilators may cause complication during ventilation, called ventilator-induced lung injury [31]. To avoid that, several innovative technical functionalities have been improved, including trigger mechanisms, apnea alarm, resistance and static dynamic compliance monitoring, lung recruitment tools (PV loops), higher number waveforms simultaneously displayed, and an adjustable screen for ergonomics. Regarding the complication post-ventilation, a recent prospective cohort study showed that the hospital mortality occurrence is related to the incidence of postoperative lung complications [32]; even ventilator-associated pneumonia (VAP), which is common for pediatrics’ patients in ICU, significantly affects the morbidity and mortality of pediatric patients [33,34,35]. The evaluation of this KPI (complication post-ventilation) has been conducted considering the management of endotracheal tube cuff pressure, representing an important element for the prevention of ventilator-associated pneumonia in patients receiving mechanical ventilation.

Some studies showed that endotracheal intubation-related complications are likely to occur in intubated pediatric patients [36]. This issue might be avoided if the ventilator model is able to optimize the NIV (non-invasive ventilation) operation, which is increasingly being used in pediatric units [37]. Some studies showed that the use of NIV in pediatric patients provides a rapid improvement of the respiratory acute injury, avoiding the requirement for endotracheal intubation reducing the related complications [38,39].

In relation to the technological risks indicator, based on our analysis, the “alerts for ventilators parameters” resulted to be the most important one. It could be observed, according to the literature and even after the evaluation process, that patient safety could be, even fatally, compromised, when a situation requiring an alarm activation is not recognized by the ventilator [37]. The “technical alarms” KPI is referred to the potential issues arising from the ventilator’s malfunctions [40] which might be overcome by the availability of programmable alarms. Ventilators also may have safety mechanisms including a control system for involuntary ventilator settings changes, calibration of flow and oxygen sensors, and RFID technology [37].

The homogeneity of technologies resulted in a pivotal aspect in the evaluation. Ensuring that as many ventilators as possible belong to an identical make and model reduces the learning curve of operators as well as the risk of errors. Finally, while choosing a ventilator model, it is important to be aware of the related adverse events and/or recalls. These aspects are evaluated into the “other technological risks for patients”.

Comparing the performances of the four ventilators models with respect of these safety indicators, results showed that Model 2 seems to be the safest one between the four technologies compared.

Evidence helped us to find out more about the clinical effectiveness domain and its indicators. Its Lev-2 KPIs are: “customization of ventilation”, “patients’ comfort”, “reduction of ventilation time”, “reduction of weaning time”. According to the studies gathered and based on the assessment process, a customized patient–ventilator interface may be useful in long-term ventilation, improving the patient–machine interaction. A number of advanced ventilation modes and innovative technical specifications (resistance and static dynamic compliance monitoring, trigger mechanism, lung recruitment tools (PV loops), programmability of frequency and amplitude of sigh breath function) guarantee the customization of ventilation. Optimizing the comfort for the patient represents also an important goal. It was also shown that the improvement of patient/device interaction is pivotal for better patient comfort as well as for the reduction of ventilation time [41]. The duration of mechanical ventilation has to be as short as possible and to this end, in the case of a neonatal patient, the ventilator system, throughout the variable support pressure ventilation mode, should provide a synchronous interaction with the infant [42]. The optimization of the duration of ventilation time might reduce the incidence of post-operative complications and can improve patients’ clinical outcomes [42]. Finally, the reduction of weaning time is another important aspect to be considered in the choice of the most appropriate ventilator model. Ventilators with automated weaning modes can overcome the problem that clinicians might under-recognize the patient’s ability to breathe without assistance, prolonging ventilation time and increasing the complications incidence [2].

The technical characteristics domain has been characterized by several technical specifications gotten from the different devices’ manufactures and from some websites such as ECRI and similar others. Technology features refer to the ventilation parameters visualization (adjustable screen, the possibility to simultaneously visualize more than one curve or loop, ease to visualize information during ventilation) and to other technical aspects such as the availability of ventilators accessories and consumables such as nebulizer, the possibility to export data to electronic health records (EHR) as well as accuracy and the availability of a wide measure range of monitored parameters. Moreover, also the management of the technology plays a crucial role, considering the importance attributed to the post-sales service, maintenance, and the training course for the users.

In relation to the cost and economic evaluation, all business proposals have been analyzed. In this way, it was possible to make an evaluation of the economic trend relative to the purchase of all consumables for the use of the device. The analysis of total cost of ownership has been carried out [29]. A detailed analysis of all service intervals and preventive maintenance requirements for each ventilator model was carried out. It was also looked at for pricing information for consumables, repair parts, preventive maintenance kits. Based on the information collected and on discussions with companies, health professionals, economists, and biomedical experts, estimated was the annual cost of ownership value for each ventilator. At this stage, a 10-year life-cycle cost estimate for each ventilator was calculated. Results showed that Model 2 resulted as being more expensive than the others, because of its peculiarities.

The analysis ended with the organizational aspects domain. Such analysis helped us to make a prevision of the impact that the deployment of new ventilators might have on existing healthcare systems. In this domain, it was possible to enhance two aspects strictly related to the selection of the ventilator model that best meets the hospital needs: the ventilator operating principles that affects the organizational aspects and the impact of the technology on the existing workflows. As for the former, KPIs included the “ease of use and ergonomics of the technology” (technical characteristics that make the ventilator more usable and enhance the human–machine interface aspects, affecting the mental workload), the “maintenance aspects”, the “versatility to use the technology with pediatric patients” due to the possibility to arbitrarily set the minimum and maximum Tidal Volume values and the “homogeneity of devices” related to the availability of portable and or neonatal ventilators of the same manufacturer or the availability of compatible accessories, to be used in the same department or in the whole hospital. The latter aspect referred to the availability of ICU beds and to the possibility to decrease the length of ICU stay if a reduction of the mechanical ventilation time occurred [1]. This aspect was carefully taken into account, and it was associated with the ventilator model’s availability of advanced weaning ventilation modes. However, the literature review results showed no significant evidence associated with the reduction of weaning ventilation time and/or ICU stay when these advanced ventilation modes are used [31,43].

To sum it all up, from our analysis, it can be summarized that the safety (in particular, that one relative to the patient safety) and the clinical effectiveness indicators resulted as the most important aspects.

The evaluation of the performance value relative to all parameters, as above described, has led to define a rank between the four models considered in the analysis. Model 2 reached the best performance value, followed by the other models. However, from the economic analysis and from a performances values point of view, even though Model 2 resulted as being more expensive than the others, because of its peculiarities, it may be considered as a great added value for the Hospital.

However, the AHP prioritization system as per definition is intrinsically subjective. The priorities derived from the professionals’ “subjective” judgments about the importance of the elements of the decision tree reflect the boundary conditions of the clinical setting in which the technology will be implemented. This method integrates scientific evidence, through which it is possible to assess the absolute performances of each ventilator model, with the judgements of the professionals involved aiming at understanding which KPI and/or domain is more important to consider for the specific healthcare context needs. Moreover, as presented in Section 3, a substantial equivalence can be observed between Model 2 and 3 performance values: Model 2 has led, indeed, only 2.3% over Model 3. To test the stability and the robustness of the final result on the possible variability of judgements, sensitivity analysis was carried out, aiming at calculating if a variation in judgements (and the entity of this variation) causes changes in the final result. It emerges that the final ranking is not robust, because with a minimum parameter variation (3% in safety performances and 5% in clinical effectiveness performances), the final decision can be inverted, confirming a substantial equivalence of the two products. From the weight system point of view, instead, results revealed a substantial stability of the final ranking. It means that the weight system elaborated is robust and coherent with the decision problem even if some changes occurred in the professionals’ judgements on the relative importance of the evaluation criteria.

This method resulted in being particularly useful when comparing particularly innovative technologies, because it is able to integrate aspects such as safety and clinical effectiveness with technical aspects, providing a unified and reliable indication about the implementation of a new technology within a hospital setting, forecasting the possible impact on its organizational frame and the economic impact over the next years.

It provides a general overview of the most relevant features to be considered in the assessment of intensive care ventilators for pediatric patients. The outputs provided by this work could be easily shared and then used systematically by other healthcare organizations.

More specifically, this study shares relevant information about all the components of the decisional hierarchy structure (i.e., KPIs) as well as their topological arrangement within the domains.

Moreover, the complete and detailed list of the technical characteristics (Lev-3 KPIs) that provide effects within the related domain were shown, explained, and discussed. In this way, readers can understand the effects that a specific technical feature might have on a specific domain.

Thus, results can then be used (or readapted) by decision-makers from other healthcare context to assess the same or similar technologies by adopting the same hierarchy structure or by adopting the same weights systems and measuring the context specific performances values.

## 5. Conclusions

This paper has provided a general overview of the most relevant features (KPIs identified) to consider in the assessment of intensive care ventilators for pediatric patients. It has discussed the main characteristics of these technologies as well as the potential implications of introducing their different innovations (proposed by the manufacturers involved in this study) into the pediatrics wards. It also provides detailed information on the methodology used to carry out the assessment.

The results will be extremely helpful for other pediatric hospitals that have to select the intensive care ventilators that best meet their needs. Results, if accurately adapted, could be easily transferred to similar hospital contexts.

It was a great opportunity for the whole working group, even for health professionals, who, with their knowledge and experience, had the chance to use and evaluate, during an intensive trial period, all available ventilators into the pediatric wards.

Four Departments tested the four ventilator models for a period of up to three months each. The entire trial test period lasted one year and the HTA process ended six months after. Even if the entire process for the selection of the best fitting model took more than one year, hospital management considered it feasible to conduct a complex and complete HTA process in order to address the significant expected expenditure for the renewal plan of all the hospital’s ventilators.

Through this assessment process, the HTA team was able to recommend to the hospital the best model available at the present time within the technological market. Integrating evidence on safety and clinical aspects with the technical, organizational, and economic evaluation, calculating the expected expenditure over 10 years, allowed decision-makers to rationally decide to conscientiously and objectively assign the rank of best performer to Model 2.

The evidence-based approach here proposed, identifying, measuring, and/or estimating the pros and cons of innovative features of ventilators, could be considered a valuable proof of evidence that conducting an HTA process before the acquisition of a technology is essential to make the best, most rational, and most reliable decision. Through this assessment process, the project team was, finally, able to recommend to the hospital management, according to specific hospital needs, the best model currently available within the technological market.

## Figures and Tables

**Table 1:** Profile of professionals involved in the study.

N°	Professionals’ Profile	Hospital Department
1	Medical Doctor	Emergency department
6	Medical Doctors	Intensive Care Unit
4	Medical Doctors	Cardiac Intensive Care Unit
1	Medical Doctor	Hospital Health Direction
5	Biomedical Engineers	Clinical Engineering Department and Health Technology Assessment Unit
1	Health Economist	Health Technology Assessment Unit

**Table 2:** Provides detailed information and description of Lev 1 (written in bold) and Lev 2 (bullet points) KPIs. Each KPI was described by a number of tenders’ specifications, each of which is measurable and intrinsically provides an effect within the related domain.

SAFETY
** Patient safety**
COMPLICATIONS DURING VENTILATION
Adjustable screen (ergonomics)
Ventilator display configurability Flow pattern/waveform adjustment
Number waveforms simultaneously displayed
Solid line waveforms displayed
Online help
Programmable sigh breath function in frequency and amplitude
Ventilation with minute volume guaranteed
Resistance and static dynamic compliance monitored
Lung recruitment tools (PV loops)
Automated broncho aspiration, pre oxygenation, post oxygenation procedures
Inspiratory Trigger mechanism
Expiratory Trigger mechanism
Apnoea alarm
NECESSITY OF ENDOTRACHEAL INTUBATION
NIV leak compensation
Automatic leak compensation, trigger flow mechanism
Endotracheal and tracheostomy tube compensation
NIV leak calculations
COMPLICATION POST VENTILATION
Endotracheal cuff pressure control
** Technological risks**
VENTILATION PARAMETERS ALARMS
Programmable alarms
TECHNICAL ALARMS
SAFETY MECHANISMS
System control for involuntary ventilator settings change
Confirmation ventilator settings
Calibration flow and oxygen sensors
RFID technology
HOMOGENEITY OF TECHNOLOGIES
OTHER TECHNOLOGICAL RISKS FOR PATIENTS
Adverse events
**CLINICAL EFFECTIVENESS**
CUSTOMIZATION OF VENTILATION
Frequency and duration of recorded trends
tracheal P-V loop visualization (with ad without CO2 monitoring)
Programmable sigh breath function in frequency and amplitude
APRV independent of the ventilator cycle.
Proportional assist ventilation (PAV)
Pressure support mode (PSV)
Resistance and static dynamic compliance monitored
Bilevel positive airway pressure (BIPAP)
NIV leak compensation
Duration of the inspiratory phase
Duration of the expiratory phase
Lung recruitment tools (PV loops)
Manual and Automated P0. 1 measurement
Advanced ventilation modes
inspiratory Trigger mechanism
Expiratory Trigger mechanism
Measurement of maximal inspiratory pressure (or NIF)
NIV leak calculations
PATIENT COMFORT
NIV
REDUCTION OF VENTILATION TIME
Ventilator display configurability
Flow pattern/waveform adjustment
Variable Pressure support mode (PSV)
REDUCTION OF WEANING TIME
Automated weaning systems
**COSTS AND ECONOMIC EVALUATION**
TOTAL COST OF OWNERSHIP
Adjustable screen (ergonomics)
Automated weaning systems
High flow oxygen therapy
Nebulization systems integrated into ventilators
Purchase cost
Consumables
Replacement parts
Technical assistance
**TECHNICAL CARACTERISTICS**
** Features of technology**
PARAMETERS VISUALIZATION
Adjustable screen (ergonomics)
TECHNICAL PERFORMANCES
Nebulization systems integrated into ventilators
Automated broncho aspiration, pre-oxygenation, post oxygenation procedures
Data export to CCE
Accuracy of ventilator parameters measurements (tidal volume, respiratory rate fraction of inspired oxygen, etc.)
** Management**
POST-SALES SERVICES
Preventive maintenance frequency
Clinical assistance
Technical assistance
TRAINING COURSE FOR OPERATORS
Support service and training maintenance course for operators
**ORGANIZATIONAL ASPECTS**
** Operating principles of technology**
EASE OF USE AND ERGONOMICS OF TECHNOLOGY
Adjustable screen (ergonomics)
Online help
Measurement of percentage levels of oxygen
Calibration flow and oxygen sensors
NIV leak compensation
Automated broncho aspiration, pre-oxygenation, post oxygenation procedures
High flow oxygen therapy
Data export to CCE
Ease of use for main features controls
Screenshot download
Nebulization systems integrated into ventilators
Preventive maintenance frequency
Support and training maintenance service
Clinical assistance
Technical assistance
Learning curve
Sterilization management
First level maintenance (set up, sterilization)
MAINTENANCE ASPECTS
VERSATILITY TO USE THE TECHNOLOGY WITH PEDIATRIC PATIENTS
Tidal Volume min and max
HOMOGENEITY OF DEVICES
Availability of transport ventilator with compatible accessories
Availability of Neonatal ICU ventilators and/or compatible accessories
** Impact on existing healthcare system**
AVAILABILITY OF BEDS

**Table 3:** List of Indicators gathered from literature review, with the relative global weights and performances percentages’ scores. Domains are listed in capital bold, Lev-1 KPIs in bold and Lev-2 KPIs in plain text.

	Performance Model 1	Performance Model 2	Performance Model 3	Performance Model 4	Weights
**SAFETY**	**36.52%**	**42.36%**	**41.93%**	**28.66%**	**45.95%**
**Patient safety**	26.15%	30.04%	29.85%	20.53%	32.40%
Complications during the ventilation	13.07%	17.98%	15.87%	10.73%	18.02%
Necessity of endo-tracheal intubation	3.38%	4.68%	4.32%	2.83%	4.68%
Complications post-ventilation	9.70%	7.37%	9.66%	6.97%	9.70%
**Technological Risks**	10.37%	12.32%	12.09%	8.13%	13.55%
Alerts for ventilators’ parameters	3.12%	4.39%	3.94%	2.90%	4.39%
Technical alerts	1.31%	1.03%	1.78%	0.56%	1.78%
Safety mechanisms	2.58%	3.52%	3.02%	1.99%	3.55%
Homogeneity of technology	2.69%	2.71%	2.23%	2.23%	2.71%
Other technological risks for the patient	0.67%	0.67%	1.12%	0.45%	1.12%
**CLINICAL EFFECTIVENESS**	**23.93%**	**30.29%**	**29.06%**	**20.00%**	**31.47%**
Customization of ventilation	6.47%	8.44%	7.75%	5.51%	8.61%
Patients comfort	4.15%	6.21%	5.01%	3.77%	6.21%
Reduction of ventilation time	5.67%	7.94%	7.77%	4.32%	8.12%
Reduction of weaning time	7.64%	7.70%	8.53%	6.40%	8.53%
**COSTS**	**2.79%**	**1.03%**	**1.24%**	**5.27%**	**5.27%**
**Total Cost of Ownership**	2.79%	1.03%	1.24%	5.27%	5.27%
**TECHNICAL CHARACTERISTICS**	**6.42%**	**7.44%**	**6.79%**	**4.45%**	**7.80%**
**Features of technology**	1.85%	2.61%	2.23%	1.59%	2.61%
Parameters visualization	0.94%	1.42%	1.18%	0.80%	1.42%
Technical performances	0.91%	1.19%	1.04%	0.80%	1.19%
**Management of the technology**	4.57%	4.83%	4.57%	2.86%	5.19%
Post-sales service	1.97%	2.23%	1.97%	1.30%	2.60%
Training course for operators	2.60%	2.60%	2.60%	1.56%	2.60%
**ORGANIZATIONAL ASPECTS**	**7.08%**	**8.65%**	**8.47%**	**5.66%**	**9.51%**
**Operating principles of technology**	4.02%	5.57%	5.06%	3.10%	6.10%
Ease of use and ergonomics of technology	1.32%	1.76%	1.55%	1.04%	1.81%
Maintenance aspects	0.43%	0.63%	0.55%	0.41%	0.63%
Versatility to use the technology with pediatric patients	1.79%	2.85%	2.46%	1.50%	2.85%
Homogeneity of devices	0.47%	0.33%	0.50%	0.16%	0.81%
**Impact on existing healthcare system**	3.06%	3.08%	3.41%	2.56%	3.41%
Availability of beds	3.06%	3.08%	3.41%	2.56%	3.41%
**TOTAL PERFORMANCE SCORES**	**76.74%**	**89.77%**	**87.49%**	**64.04%**	

**Table 4:** Economic evaluation: Total Cost of ownership, hypothesizing 10-year life-cycle cost estimate for each ventilator.

	Model 1	Model 2	Model 3	Model 4
Purchase cost	€25,479.24	€27,414.91	€23,395.08	€13,783.34
Replacement parts	€1750.00	€5052.00	€5686.90	€5198.00
Consumable items	-	€5395.50	-	-
Maintenance Costs (Full Risk)	€10,701.28	€9650.05	€14,972.85	€11,761.32
TOTAL COST OF OWNERSHIP	€37,930.53	€47,512.46	€44,054.83	€30,742.66

**Figure A2:**
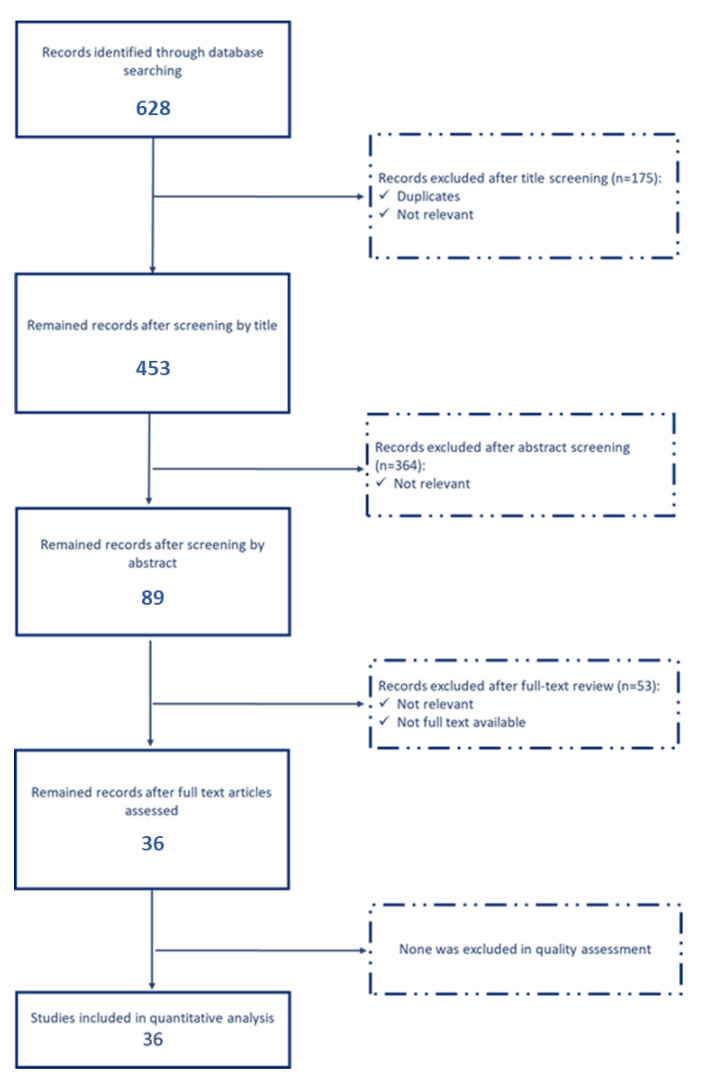
Screening process.

## Data Availability

The datasets used and analyzed during the current study are available from the corresponding author on reasonable request.

## Appendix B

A general literature search was carried out aimed at collecting all the relevant aspect for the assessment of intensive care ventilators in order to outline a clear, detailed, and proper definition of the KPIs, answering the questions of the selected assessment elements (see EunetHTa CoreModel). The search was not limited to scientific papers (Pubmed) but included also gray literature (Google), white papers (ECRI), and manufacturers’ websites in order to gather as much evidence as possible on the safety, clinical effectiveness, costs, organizational and technical aspects of the technologies under evaluation.

The keywords used for the search were: “Ventilators Mechanical”, “Respiration”, “Artificial”, “Adverse effects”, “Complications”, “Mortality”, “Instrumentation”, “Ventilator Weaning”, “length of stay”, “Children”, “Child”, “Respiration, Artificial/adverse effects” (Mesh), “Respiration, Artificial/ complications” (Mesh), “Respiration, Artificial/mortality” (Mesh), “Respiration, Artificial/ instrumentation” (Mesh), “Ventilator Weaning” (Mesh). The combination of the keywords, and the search engine used varied with the specific domain, topic, and issue we were searching information for.

Considering only the Pubmed search, a total of 628 articles were identified, of which 36 were considered relevant for the final scope of identifying the KPIs able to assess the performance of intensive care ventilators. The Figure A2 describes the screening process.
